# Molecular Links Between Circadian Rhythm Disruption, Melatonin, and Neurodegenerative Diseases: An Updated Review

**DOI:** 10.3390/molecules30091888

**Published:** 2025-04-23

**Authors:** Kemal Hüsnü Can Baser, Ismail Celil Haskologlu, Emine Erdag

**Affiliations:** 1 Department of Pharmacognosy, Faculty of Pharmacy, Near East University, 99138 Nicosia, Cyprus; 2 Department of Pharmacology, Faculty of Pharmacy, Near East University, 99138 Nicosia, Cyprus; ismailcelil.haskologlu@neu.edu.tr; 3 Department of Pharmaceutical Chemistry, Faculty of Pharmacy, Near East University, 99138 Nicosia, Cyprus

**Keywords:** circadian rhythm, clock genes, melatonin, neurodegenerative diseases

## Abstract

Circadian rhythms are molecular oscillations governed by transcriptional–translational feedback loops (TTFLs) operating in nearly all cell types and are fundamental to physiological homeostasis. Key circadian regulators, such as circadian locomotor output cycles kaput (CLOCK), brain and muscle ARNT-like 1 (*BMAL1*), period (*PER*), and cryptochrome (*CRY*) gene families, regulate intracellular metabolism, oxidative balance, mitochondrial function, and synaptic plasticity. Circadian disruption is known as a central contributor to the molecular pathophysiology of neurodegenerative disorders. Disease-specific disruptions in clock gene expression and melatoninergic signaling are known as potential early-stage molecular biomarkers. Melatonin, a neurohormone secreted by the pineal gland, modulates clock gene expression, mitochondrial stability, and inflammatory responses. It also regulates epigenetic and metabolic processes through nuclear receptors and metabolic regulators involved in circadian and cellular stress pathways, thereby exerting neuroprotective effects and maintaining neuronal integrity. This review provides recent findings from the past five years, highlighting how circadian dysregulation mediates key molecular and cellular disturbances and the translational potential of circadian-based therapies in neurodegenerative diseases.

## 1. Introduction

The field of chronobiology garnered increasing scientific attention, particularly following the recognition of key discoveries related to the molecular mechanisms that regulate circadian rhythms with the 2017 Nobel Prize in Physiology or Medicine [1]. This recognition emphasized the biological significance of internal clocks and paved the way for translational research linking circadian disruption to complex human diseases, including neurodegeneration [2]. Circadian rhythms are intrinsic biological cycles that follow an approximately 24 h period, regulating a wide array of biological activities and psychological functions, involving processes like the regulation of sleep timing, hormonal balance, metabolic activity, and cognitive function. These rhythms are mediated by transcriptional–translational feedback loops (TTFLs), in which core clock genes generate oscillatory patterns of gene expression that synchronize cellular functions across different tissues [3,4,5].

**Figure 1:**
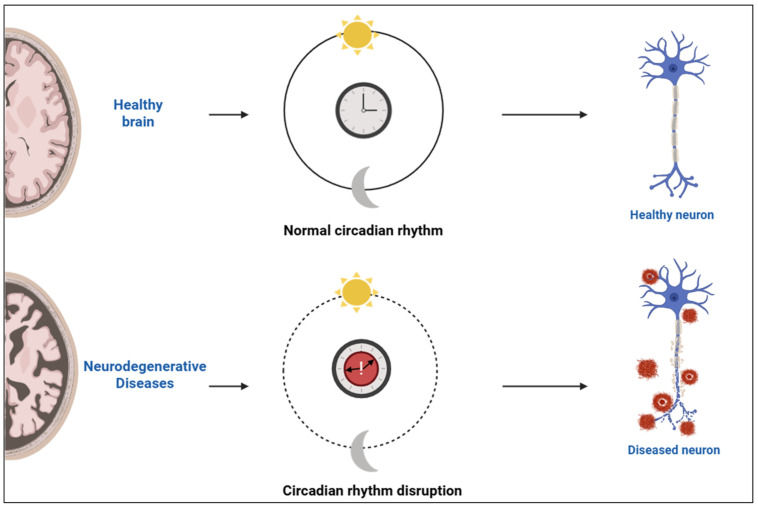
Schematic representation of circadian rhythm integrity and its impact on neuronal health. Created with https://www.biorender.com/ (accessed on 17 April 2025).

At the systemic level, the suprachiasmatic nucleus (SCN) of the hypothalamus functions as the primary coordinator of circadian rhythms, aligning peripheral clocks located throughout the body. Light input from the retina entrains the SCN, ensuring synchronization with external environmental cues [6]. This alignment between central and peripheral clocks is crucial for physiological homeostasis, especially within the central nervous system, where circadian timing tightly regulates neuronal excitability, synaptic plasticity, and energy metabolism. Under normal conditions, coordinated circadian oscillations support healthy brain structure and neuronal function. Disruption of these rhythms, commonly observed in neurodegenerative diseases, leads to loss of synchrony and impaired neuronal activity [6,7]. A conceptual illustration of this rhythmic regulation and its disruption in neurodegenerative states is depicted in Figure 1.

An increasing number of clinical studies suggest that circadian rhythm disturbances are prevalent across multiple neurodegenerative diseases. Common manifestations such as fragmented sleep, altered melatonin secretion, disrupted core body temperature rhythms, and irregular motor activity patterns are documented in both the early and advanced stages of these diseases [8,9]. Notably, these disruptions often occur before the onset of major neurological symptoms, suggesting that circadian misalignment may serve as an early biomarker and contributing factor to disease pathogenesis [10].

Among the principal regulators of circadian timing is melatonin. While widely recognized for its role in promoting sleep, melatonin also exerts antioxidant, anti-inflammatory, and mitochondria-stabilizing effects [11]. These pleiotropic actions make melatonin an increasingly relevant molecule in the context of neurodegenerative disease research.

As a key hormonal mediator downstream of the core circadian clock machinery, melatonin functions as a molecular bridge that conveys temporal information to peripheral systems. Its rhythmic secretion under SCN control translates internal clock dynamics into systemic physiological regulation. In the past five years, research has increased around the molecular interplay between circadian regulation, melatoninergic signaling, and neurodegenerative processes. Yet, existing literature remains largely fragmented and disease-specific [12,13,14,15,16,17,18,19]. Most prior reviews have focused on individual diseases or limited aspects of circadian biology, lacking a comprehensive framework that unites molecular clock mechanisms, hormonal mediators like melatonin, and their relevance to disease progression.

This review aims to fill that gap by providing an integrative and up-to-date synthesis of recent findings, highlighting converging molecular pathways, shared mechanistic disruptions, and clinically relevant chronobiological targets. Emphasis is placed on the translational potential of circadian-based interventions, including both pharmacological and lifestyle-oriented strategies, for improving early diagnosis, therapeutic precision, and quality of life in patients with neurodegenerative disorders.

## 2. Molecular Basis of Circadian Rhythms

### 2.1. Core Clock Genes and Mechanisms

In mammals, the central circadian rhythm regulator is the SCN, which resides in the anterior part of the hypothalamus. Composed of approximately 20,000 neurons, the SCN generates self-sustaining rhythmic signals that synchronize peripheral clocks throughout the body [20]. Photic signals are transmitted to the SCN via the retinohypothalamic tract, a monosynaptic pathway derived from photosensitive retinal ganglion cells (ipRGCs) that detect ambient light and convey circadian information to the brain. This light input activates intracellular signaling pathways in SCN neurons, triggering calcium influx and phosphorylation of cAMP response element-binding protein (CREB), thereby inducing clock gene expression [21].

SCN neurons exhibit intrinsic circadian oscillations in membrane excitability and neuropeptide release. Key signaling molecules such as vasoactive intestinal peptide (VIP) and arginine vasopressin (AVP) help maintain synchronization within the SCN and transmit time cues to peripheral tissues. The SCN exerts systemic control via neuroendocrine pathways, autonomic innervation, and behavioral regulators, including circadian-driven activity patterns and feeding [22].

Peripheral circadian oscillators are distributed throughout almost all bodily tissues and follow a similar transcriptional framework. However, unlike the SCN, they are more strongly influenced by non-light-related signals such as nutrient intake, thermal conditions, and glucocorticoid levels. The interplay between central and peripheral oscillators is crucial for maintaining circadian coherence and physiological homeostasis [23].

While the SCN orchestrates systemic timing, the fundamental rhythm of each cell is governed by molecular feedback loops known as TTFLs. The positive limb of the core loop is driven by CLOCK and BMAL1 transcription factors, which dimerize and interact with E-box sequences located within the promoter domains of downstream genes. This interaction initiates the expression of *Period* (*PER1*, *PER2*, *PER3*) and *Cryptochrome* (*CRY1*, *CRY2*) genes, forming the inhibitory branch of the loop [24].

After translation, PER and CRY proteins undergo post-translational modifications, particularly phosphorylation by Casein Kinase 1δ/ε (CK1δ/ε), which regulates their stability, nuclear translocation, and function. Inside the nucleus, the PER–CRY complexes inhibit CLOCK–BMAL1 activity, establishing a classic negative feedback mechanism that completes approximately every 24 h [25].

A secondary interlocking loop involving REV-ERBα and retinoic acid receptor-related orphan receptor alpha (RORα) further stabilizes the rhythm by modulating *BMAL1* transcription through RREs (ROR response elements). RORα acts as a transcriptional activator, while REV-ERBα serves as a repressor, maintaining amplitude and phase consistency in circadian gene expression [24,25]. The central and peripheral circadian regulators, along with the roles of core clock genes in TTFLs, are summarized in Table 1.

**Figure 2:**
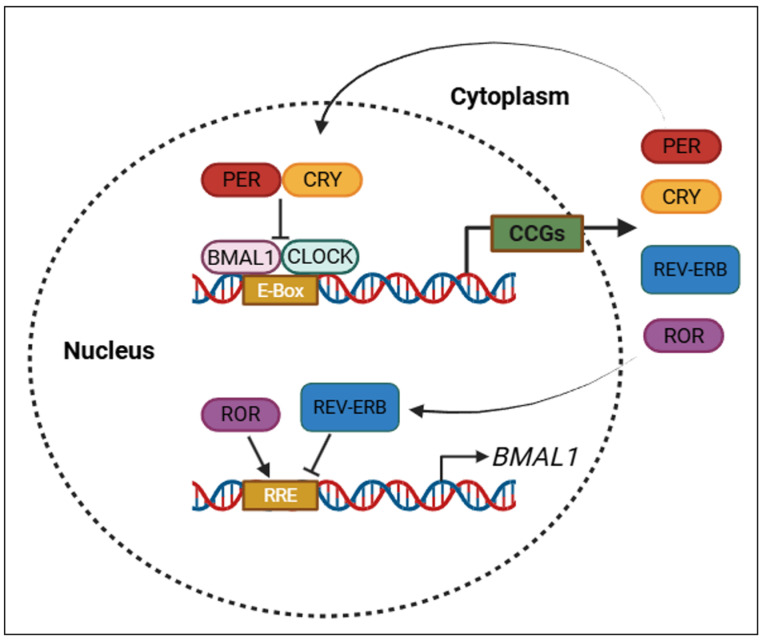
Core transcriptional–translational feedback loops (TTFLs) that regulate circadian rhythms. PER: period; CRY: cryptochrome; BMAL1: brain and muscle ARNT-like protein-1; CLOCK: circadian locomotor output cycles kaput; REV-ERB: nuclear receptor subfamily 1 group D member 1; ROR: retinoic acid-related orphan receptor; CCGs: clock-controlled genes; E-box: enhancer box (DNA sequence bound by CLOCK–BMAL1); RRE: ROR response element. Created with https://www.biorender.com/ (accessed on 17 April 2025).

As illustrated in Figure 2, TTFLs ensure the rhythmic expression of circadian clock genes through coordinated transcriptional activation and feedback repression mechanisms.

### 2.2. Molecular and Cellular Targets of Circadian Clock Genes

Circadian clocks govern various physiological functions such as metabolism, oxidative balance, mitochondrial function, and synaptic plasticity. For instance, BMAL1 was shown to influence glucose homeostasis, lipid metabolism, and the rhythmic expression of metabolic enzymes. Dysregulation of BMAL1 impairs mitochondrial biogenesis and leads to increased reactive oxygen species (ROS) production, thereby contributing to cellular stress [26].

In the central nervous system, clock genes also regulate synaptic activity, neuronal excitability, and neurotransmitter release. For instance, *BMAL1* deficiency impairs synaptic vesicle cycling and increases susceptibility to oxidative damage and inflammation, indicating that circadian control is vital for maintaining neuronal function and plasticity [26,27].

In addition to these systemic and neurophysiological roles, circadian rhythms also fine-tune fundamental cellular processes such as apoptosis, autophagy, DNA repair, and cell cycle regulation. These cellular pathways follow time-of-day-dependent patterns and are directly modulated by clock gene activity [28,29]. For example, the circadian regulation of autophagy ensures optimal clearance of damaged organelles and misfolded proteins during rest phases—an especially important mechanism in neurodegenerative contexts where protein aggregation and mitochondrial dysfunction are key pathological features [30].

Furthermore, multiple epigenetic and regulatory layers, including histone modifications, non-coding RNAs, chromatin remodeling, and metabolic feedback loops, have emerged as integral components of circadian regulation. These additional mechanisms increase the adaptability of the molecular clock and enhance its responsiveness to environmental cues such as light, nutrition, and stress [31].

### 2.3. Melatonin and Other Hormonal Regulators

Melatonin, which is predominantly released by the pineal gland at night, is crucial for stabilizing biological rhythms and aligning peripheral clocks with the SCN. Melatonin is synthesized through a multi-step pathway starting with tryptophan, an essential amino acid. This precursor undergoes hydroxylation to form 5-hydroxytryptophan, followed by a decarboxylation step resulting in serotonin production [32,33]. Subsequently, serotonin is converted via acetylation by the enzyme arylalkylamine N-acetyltransferase (AANAT), which acts as a key regulatory step in the process. The activity of AANAT is tightly regulated by noradrenergic stimulation from the SCN and converted to melatonin via hydroxyindole-O-methyltransferase (HIOMT), also known as ASMT (acetylserotonin O-methyltransferase). The light-dark cycle, through retinal input to the SCN, modulates the sympathetic output to the pineal gland and ultimately dictates melatonin synthesis [34,35].

Melatonin exerts its physiological actions through two high-affinity G-protein-coupled receptors, MT1 (melatonin receptor 1A) and MT2 (melatonin receptor 1B), which are widely expressed in both central and peripheral tissues. MT1 is primarily involved in the acute suppression of neuronal firing in the SCN, contributing to sleep promotion and phase regulation. Conversely, MT2 is significantly involved in adjusting circadian timing and contributes notably to the realignment of misaligned biological clocks [36,37].

Beyond their canonical roles in regulating circadian rhythms, melatonin receptors MT1 and MT2 initiate several intracellular signaling cascades that are critically involved in the molecular pathogenesis of neurodegenerative diseases. One key pathway involves phosphatidylinositol 3-kinase (PI3K) and protein kinase B (AKT), where receptor activation leads to phosphorylation and subsequent inhibition of glycogen synthase kinase-3 beta (GSK3β). GSK3β is a serine/threonine kinase implicated in tau protein hyperphosphorylation and amyloid-beta (Aβ) accumulation, both of which are hallmarks of Alzheimer’s disease (AD) pathology [38].

In parallel, melatonin upregulates the expression and activity of sirtuin 1 (SIRT1), a nicotinamide adenine dinucleotide (NAD^+^)-dependent deacetylase. SIRT1 enhances mitochondrial biogenesis by activating peroxisome proliferator-activated receptor gamma coactivator 1-alpha (PGC-1α) and contributes to chromatin remodeling, circadian gene regulation, and resistance to cellular stress in neurons. Additionally, melatonin was shown to suppress the activation of the NOD-like receptor protein 3 (NLRP3) inflammasome, a multiprotein complex involved in innate immune signaling and chronic neuroinflammation. By inhibiting NLRP3, melatonin mitigates oxidative damage and glial overactivation [38].

In aging and several neurodegenerative disorders, melatonin secretion is reduced, and receptor responsiveness is impaired. This dysregulation contributes to circadian desynchronization, disrupted cellular homeostasis, and progressive neurodegeneration [39,40]. Understanding how melatonin signaling intersects with disease-relevant molecular targets offers promising opportunities for therapeutic intervention.

**Figure 3:**
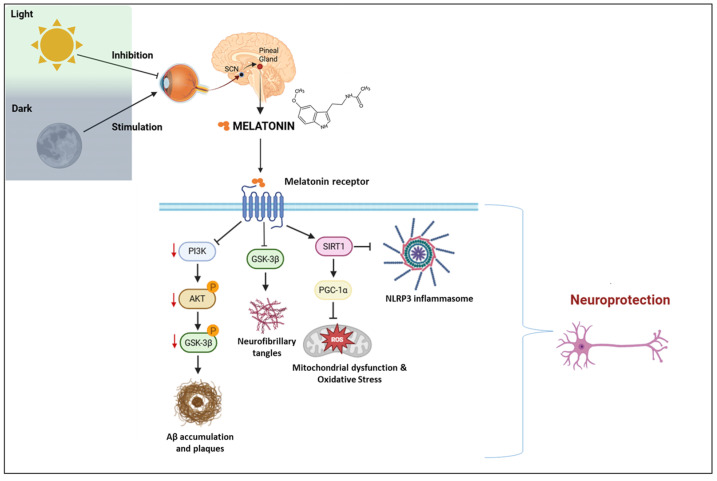
Melatonin signaling pathways and neuroprotective mechanisms in the context of circadian regulation. SCN: suprachiasmatic nucleus; PI3K: phosphoinositide 3-kinase; AKT: protein kinase B; GSK3β: glycogen synthase kinase 3 beta; Aβ: amyloid-beta; SIRT1: sirtuin 1; PGC-1α: peroxisome proliferator-activated receptor gamma coactivator 1-alpha; NLRP3: NOD-like receptor family pyrin domain-containing 3; ROS: reactive oxygen species. Created with https://www.biorender.com/ (accessed on 17 April 2025).

The receptor-mediated molecular pathways of melatonin are illustrated in Figure 3, which integrates circadian signaling with neurodegenerative mechanisms such as tau hyperphosphorylation, oxidative stress, and neuroinflammation.

### 2.4. Melatonin and Blood–Brain Barrier Integrity

Melatonin was shown to exert a protective influence on the blood–brain barrier (BBB), acting through a range of molecular pathways that preserve barrier function and structural integrity. One of its key mechanisms involves the activation of melatonin receptors, which enhances the activity of P-glycoprotein transporters, crucial components for regulating the efflux of neurotoxic substances [41,42,43,44]. This effect was particularly noted in in vivo experimental rat models of methamphetamine-induced toxicity, where melatonin prevented endothelial damage in brain capillaries by inhibiting NADPH oxidase 2 through receptor-mediated pathways [45,46,47,48].

Evidence from various preclinical models further supported melatonin’s role in maintaining BBB integrity. In in vivo neonatal rat models subjected to excitotoxic insults, melatonin significantly reduced BBB disruption. Likewise, in young C57BL/6 mouse models exposed to transient focal cerebral ischemia, melatonin administration attenuated vascular leakage and preserved barrier function, underscoring its potential as a neurovascular protector in ischemic and excitotoxic conditions [49,50].

At the molecular level, melatonin contributes to the maintenance of tight junction integrity by upregulating proteins such as claudin-5, zonula occludens-1 (ZO-1), and occludin—key structural components that prevent paracellular diffusion of harmful blood-borne substances into the brain parenchyma [51]. In in vitro models using human brain microvascular endothelial cells (hBMECs), melatonin was shown to increase the expression of these tight junction proteins and strengthen barrier integrity.

In addition, melatonin may interfere with angiotensin-converting enzyme 2 (ACE2), a membrane receptor facilitating viral entry into brain endothelial cells. Experimental findings indicated that consistent use of melatonin and related melatonergic compounds can reduce ACE2-dependent neuroinvasion by SARS-CoV-2, which is notable given the virus’s potential to intensify Aβ toxicity and oxidative burden in AD patients [52].

Moreover, melatonin modulates the activity of matrix metalloproteinases (MMPs), enzymes known to compromise BBB stability by degrading extracellular matrix proteins [53,54]. In vitro experiments conducted on human gastric adenocarcinoma (AGS) cell lines indicated that melatonin decreases MMP activity, suggesting a broader role in extracellular matrix regulation.

In aged (18–20-month-old) C57BL/6 mouse models challenged with lipopolysaccharide to induce neuroinflammation and increased BBB permeability, melatonin activated AMP-activated protein kinase (AMPK) in endothelial cells, an essential kinase involved in homeostasis and vascular integrity. These findings collectively point to melatonin as a potent modulator of BBB function, with therapeutic implications in conditions characterized by vascular dysfunction and neuroinflammation [55,56,57,58]. Table 2 presents preclinical evidence demonstrating how melatonin modulates key molecular mechanisms to protect the BBB.

## 3. Circadian Disruption as a Pathophysiological Trigger

### 3.1. Environmental and Lifestyle Factors

In modern society, circadian disruption is prevalent due to environmental and lifestyle-related factors. Exposure to artificial light at night (ALAN), irregular sleep–wake cycles, shift work, and frequent transmeridian travel (jet lag) can profoundly disturb endogenous circadian rhythms. These external cues, or “zeitgebers”, are crucial for entraining the central and peripheral clocks, and their disruption leads to a desynchronization between internal physiological processes and the external environment [59,60,61,62]. Night-shift workers, for instance, exhibit a significantly higher prevalence of metabolic syndrome, mood disturbances, and cognitive impairment—conditions also associated with circadian misalignment and neurodegenerative vulnerability [63]. Moreover, chronic exposure to ALAN was shown to suppress nocturnal melatonin production, disrupt sleep architecture, and interfere with core body temperature rhythms, leading to increased oxidative stress and neuroinflammatory activity [64]. A recent experimental model further supported these findings, demonstrating that artificial light exposure induces hippocampal neuronal loss and impairs memory consolidation in rodents [65]. These results highlighted the potential neurobiological consequences of persistent lifestyle-related chronodisruption and suggested its role as a modifiable environmental risk factor for neurodegenerative disease.

### 3.2. Chronodisruption and Aging

Aging is intrinsically associated with the progressive attenuation of circadian rhythms, both behaviorally and at the molecular level. Age-related changes in the SCN include reduced neuronal synchronization, impaired responsiveness to light cues, and diminished expression of core clock genes such as *BMAL1*, *PER2*, and *CRY1*. These molecular alterations contributed to weakened circadian amplitude and phase shifts, often manifested as fragmented sleep, advanced sleep phase syndrome, and reduced melatonin secretion [66]. Importantly, aging also affects the epigenetic regulation of clock genes, including changes in DNA methylation and histone acetylation patterns, which may compromise the plasticity of circadian responses [67]. Disruption of circadian rhythms amplifies critical aging-related impairments such as impaired mitochondrial performance, elevated reactive oxygen species production, and persistent low-level inflammatory responses. This creates a feedback loop that accelerates neurodegenerative processes. Recent transcriptomic analyses of aged brain tissue revealed a global dampening of circadian gene oscillations in both neurons and glial cells, implicating circadian breakdown as a systemic hallmark of brain aging [68].

In aged human prefrontal cortex tissue, the amplitude and rhythmicity of core clock genes such as *BMAL1* and *PER2* were shown to decrease significantly. A transcriptomic study involving 146 human samples revealed over 1000 transcripts with age-related changes in circadian patterns, including marked reductions in *BMAL1* and *PER2* expression. In the SCN and peripheral oscillators, aging was found to impair intracellular coupling and attenuate circadian output [69]. Additionally, bioluminescence imaging of *PER2::LUC* rhythms revealed a significant reduction in oscillatory strength in aged SCN tissue, indicating impaired synchrony among SCN neurons. Although core molecular oscillations may persist, the reduced coupling efficiency compromises the SCN’s ability to coordinate peripheral clocks, contributing to systemic circadian disruption observed with aging [70].

### 3.3. Circadian and Sleep-Based Biomarkers in Neurodegeneration

Given the mounting evidence linking circadian dysregulation with neurological decline, there is growing interest in identifying circadian biomarkers for early detection, disease staging, and therapeutic monitoring in neurodegenerative disorders. Core clock genes, particularly *BMAL1*, *PER2*, and REV-ERBα, exhibited disease-specific alterations in expression patterns in both central and peripheral tissues of patients with AD, Parkinson’s disease (PD), and Huntington’s disease (HD) [10,71]. Expression of peripheral clock genes in blood cells or buccal epithelium showed potential as a non-invasive biomarker indicating central circadian disruptions. In AD, diurnal fluctuations in melatonin levels and rest-activity rhythms correlate with cognitive decline and were proposed as prognostic indicators of disease progression [72,73].

Sleep is essential for preserving brain homeostasis, particularly through its facilitation of glymphatic clearance, a process in which cerebrospinal fluid (CSF) flows through perivascular spaces to remove metabolic waste, including neurotoxic proteins such as amyloid-beta (Aβ), tau, and α-synuclein. Sleep disruptions, particularly reductions in slow-wave sleep and fragmentation of the sleep–wake cycle, impair glymphatic function and contribute to the accumulation of these pathological proteins, thereby accelerating neurodegenerative processes [74,75]. Circadian dysfunction often manifests early as REM sleep behavior disorder (RBD), a condition now recognized as a predictive biomarker for neurodegenerative diseases such as PD and Lewy body dementia [76]. Moreover, wearable devices that track actigraphy and body temperature rhythms are emerging as potential digital tools to monitor circadian integrity longitudinally. Advances in machine learning and integrative chronobiomics further enable the characterization of individualized circadian profiles, offering new opportunities for personalized chronotherapeutic interventions [77]. These approaches hold the potential to improve diagnostic accuracy and therapeutic timing, ultimately enhancing outcomes in patients with neurodegenerative diseases.

## 4. Neurodegenerative Diseases and Circadian Dysregulation

**Figure 4:**
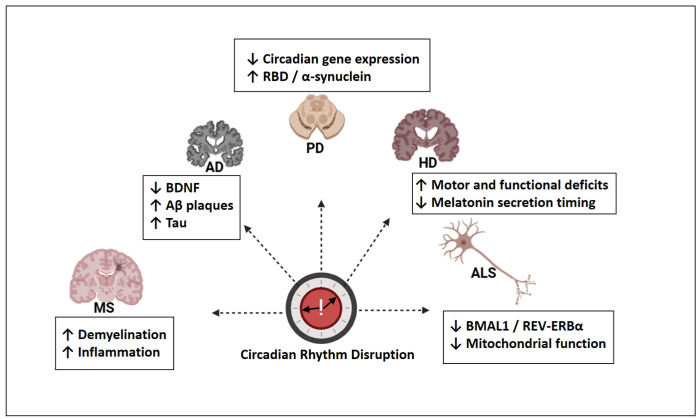
Disease-specific patterns of circadian rhythm disruption in major neurodegenerative disorders. AD: Alzheimer’s disease; PD: Parkinson’s disease; HD: Huntington’s disease; ALS: amyotrophic lateral sclerosis; MS: multiple sclerosis; BDNF: brain-derived neurotrophic factor; Aβ: amyloid-beta; Tau: microtubule-associated protein tau; RBD: REM sleep behavior disorder; BMAL1: brain and muscle ARNT-like 1; REV-ERBα: nuclear receptor subfamily 1, group D, member 1; ↓ = decreased; ↑ = increased. Created with https://www.biorender.com/ (accessed on 17 April 2025).

Circadian rhythm disruption presents both convergent and disorder-specific alterations across neurodegenerative diseases. Figure 4 provides an overview of these features, which were discussed in greater detail in the subsequent sections.

### 4.1. Circadian Dysregulation and Melatonin in AD

#### 4.1.1. Circadian Rhythm Disturbances as Early Features of AD

AD, the most common form of dementia, is frequently accompanied by circadian rhythm disturbances and sleep–wake disruptions, often preceding the onset of cognitive symptoms. These alterations, characterized by fragmented sleep, reduced amplitude of circadian markers, and diminished melatonin secretion, are increasingly recognized not only as clinical manifestations but also as potential contributors to disease progression [78,79]. Notably, cerebrospinal fluid (CSF) and plasma melatonin levels are markedly reduced in AD patients, particularly during the nocturnal phase, and correlate with disease severity and disrupted activity-rest cycles [80].

Circadian disturbances in AD are associated with reduced expression of neurotrophic factors such as BDNF, contributing to synaptic dysfunction and cognitive deterioration [81]. The bidirectional interplay between circadian dysregulation and AD pathology suggested that rhythm disturbances may act as both a consequence and a driver of neurodegeneration [82]. For instance, sleep deprivation impaired glymphatic clearance of Aβ and tau, exacerbating their accumulation and plaque formation [9,83].

#### 4.1.2. Clinical Trials and Variability in Melatonin Therapy

Clinical studies further highlighted melatonin’s therapeutic potential. A randomized trial in the Netherlands involving nearly 200 elderly nursing home residents (mean age: 86; 87% with dementia) showed that a 15-month regimen combining 2.5 mg melatonin with daytime bright light therapy significantly reduced agitation and improved sleep quality [84,85]. However, the findings in the literature continued to be inconsistent. While some trials report no significant improvement in sleep among patients with advanced AD [86,87], these inconsistencies may reflect disease-stage-related neurodegeneration of circadian regulatory centers or individual genetic variability, such as polymorphisms in melatonin receptor genes (e.g., *MTNR1A*, *MTNR1B*) that affect responsiveness to exogenous melatonin [88].

Inconsistencies across clinical trials may also stem from considerable variability in melatonin dosage, administration timing (bedtime vs. chronobiologically optimized schedules), and formulation (immediate- vs. sustained-release). Moreover, patient heterogeneity regarding the stage of AD progression, baseline melatonin levels, and co-administration with light therapy or hypnotic agents may significantly influence treatment outcomes. The presence of polymorphisms in *MTNR1A* and *MTNR1B* genes further complicates the picture, as they may alter receptor sensitivity and downstream signaling. Future trials should adopt stratified designs incorporating genotypic profiling, circadian biomarker analysis, and standardized dosing protocols to better delineate responder subgroups and optimize therapeutic efficacy.

#### 4.1.3. Observational and Epidemiological Insights

The use of melatonin in the elderly population has increased significantly over the past two decades. In the U.S., its use among individuals aged 65 and older rose from 0.6% in 1999 to 2.1% in 2018, with similar trends in other countries. While generally considered safe, pharmacokinetic studies suggested that older adults may exhibit increased melatonin absorption, potentially enhancing its therapeutic effects but warranting further pharmacodynamic investigation [89].

Observational studies also linked melatonin dysregulation to increased AD risk. Misalignment between endogenous melatonin rhythms and environmental light-dark cycles was associated with elevated vulnerability to neurodegeneration. A large UK Biobank study (*n* ≈ 276,000) reported that individuals working permanent night shifts had a 1.5-fold higher risk of developing AD over a nine-year follow-up. Similarly, early-life visual impairment, reducing light input to the circadian system, was correlated with increased AD risk, underscoring the importance of environmental synchronization for brain health [90,91].

#### 4.1.4. Mechanistic and Preclinical Evidence of Melatonin

On a biochemical level, clinical assessments revealed that CSF melatonin concentrations are significantly lower in AD patients compared to age-matched controls, and these reductions were associated with increased disease severity [80,92]. Preclinical research supported that melatonin supplementation improved spatial memory and learning capacity, particularly through modulation of hippocampal CREB and BDNF signaling. Different experimental reports involving 294 rodents confirmed melatonin’s efficacy in reversing cognitive deficits, with notable improvements in Morris Water Maze performance across diverse AD models [93].

Further studies in developmental and metabolic models revealed that melatonin attenuates cognitive impairments through molecular pathways involving SIRT1, Mfn2, and PERK, indicating a role in alleviating endoplasmic reticulum stress [94]. A meta-analysis of 22 randomized controlled trials reported that melatonin administration over 12 weeks improved MMSE scores, especially in early-stage AD. Additionally, higher endogenous melatonin levels were linked to reduced cognitive impairment and depressive symptoms in community-dwelling older adults [95].

Short-term melatonin supplementation (e.g., 1 mg daily for four weeks) showed cognitive enhancement in healthy elderly individuals, improving verbal memory performance [96]. Similarly, in younger adults, melatonin was found to improve recognition memory for stress-encoded stimuli, suggesting its role in modulating emotional memory consolidation [97]. In clinical populations such as hemodialysis patients, 3 mg of melatonin nightly for six weeks led to significant improvements in MoCA scores [98,99]. Neuroimaging studies also associated higher pre-bedtime melatonin levels with increased hippocampal volume, an area central to memory and susceptible to AD pathology [100].

A growing body of research also implicated melatonin in the regulation of glymphatic function, a sleep-dependent clearance system responsible for removing interstitial waste, including Aβ. The glymphatic pathway involves CSF influx through periarterial spaces, interstitial exchange, and efflux via perivenous routes [101,102]. Aquaporin-4 (AQP4)–expressing astrocytes played a critical role in this system, and melatonin appeared to enhance its function by improving AQP4 polarization. In rodent models of chronic stress, melatonin administration restored glymphatic integrity and normalized waste clearance [102]. Moreover, melatonin’s suppression of orexin, an arousal-promoting neuropeptide, may support glymphatic efficacy by promoting deeper sleep and reducing nocturnal sympathetic activity [103]. Melatonin was also shown to lower nighttime blood pressure, further facilitating CSF–CSF-interstitial fluid exchange [104].

#### 4.1.5. Melatonin as a Biological Supplement

Considering its pleiotropic neuroprotective effects, favorable safety profile, and wide availability, melatonin holds promise as a biological supplement for patients with AD. Clinical studies and meta-analyses reported improvements in sleep quality, cognitive performance, and behavioral symptoms. Melatonin’s antioxidant, anti-inflammatory, and circadian-stabilizing properties support its potential as an adjunctive therapy [105,106]. However, clinical outcomes remain variable due to differences in dosage, formulation, treatment duration, and patient chronotype [107]. Future trials should adopt biomarker-guided and genotype-stratified approaches to better identify responders and optimize treatment protocols.

### 4.2. Circadian Dysregulation and Melatonin in PD

#### 4.2.1. Preclinical Evidence

PD is a progressive neurodegenerative disorder primarily known for its motor symptoms. However, non-motor manifestations, particularly those related to circadian rhythm and sleep architecture, are now recognized as crucial components of disease pathophysiology. Circadian disturbances frequently precede motor onset, suggesting that chronobiological dysfunction may not only reflect but also contribute to disease progression [108].

Disruption of the master circadian regulator, the SCN, leads to impaired synchronization of sleep–wake cycles, hormonal secretion, and thermoregulation. This is accompanied by reduced amplitude in the expression of essential circadian genes, along with diminished nocturnal melatonin release. Notably, a continuum of circadian rhythm degradation was observed across the spectrum of α-synucleinopathies, from isolated REM sleep behavior disorder (iRBD) to PD and dementia with Lewy bodies (DLB) [109]. These findings emphasized the relevance of circadian biomarkers in tracking disease evolution.

Furthermore, experimental models demonstrated that melatonin exerts dopaminergic neuroprotection by reducing reactive oxygen species, modulating the Nrf2 pathway, and suppressing pro-inflammatory cytokines [110]. Additionally, its interaction with REV-ERBα (NR1D1) highlighted its potential dual role in maintaining molecular circadian homeostasis and regulating neuroimmune responses [16].

#### 4.2.2. Clinical Evidence

REM sleep behavior disorder (RBD), characterized by loss of muscle atonia and vivid dream enactment, is prevalent in prodromal and early-stage PD. Since REM sleep is under strong circadian regulation, the presence of RBD may reflect a deeper chronobiological misalignment [111]. A review study conducted by Surmeier et al. emphasized that RBD is correlated with more severe dopaminergic deficits and increased α-synuclein burden [112]. The progression from RBD to PD involves increasing sleep fragmentation, daytime sleepiness, and a breakdown in circadian amplitude, all of which were linked to thalamocortical dysfunction and SCN impairment [113].

Melatonin supplementation had beneficial effects on RBD symptoms in PD, reducing dream enactment behaviors and improving REM sleep quality. Furthermore, observational studies and small clinical trials suggested that melatonin may support circadian entrainment, mitigate sleep disturbances, and improve overall quality of life in PD patients [114]. However, heterogeneity in dosage, duration, and patient characteristics necessitates further controlled trials to establish optimal treatment parameters [115].

Complementary approaches such as structured light therapy, exercise, and music therapy also demonstrated efficacy in reinforcing circadian structure and alleviating both motor and non-motor symptoms in PD. For instance, daily exposure to bright morning light enhanced sleep efficiency, shortened sleep onset times, and even enhanced motor coordination. Music-based interventions that align with individual circadian patterns were shown to improve mood and rhythmic motor performance [116].

### 4.3. Circadian Dysregulation and Melatonin in HD

#### 4.3.1. Preclinical Evidence

HD is a hereditary neurodegenerative condition inherited in an autosomal dominant pattern, marked by worsening motor impairments, declining cognitive abilities, and psychiatric symptoms. A recent preclinical study demonstrated that melatonin supplementation can restore circadian rhythmicity and alleviate behavioral abnormalities in HD models. For instance, in Drosophila models, melatonin treatment modulated circadian gene expression and improved locomotor function [117].

#### 4.3.2. Clinical Evidence

Emerging evidence suggests that circadian rhythm disruptions are not merely secondary consequences of neurodegeneration but may play a mechanistic role in HD pathophysiology [118].

Patients with HD often exhibit delayed melatonin secretion onset and altered acrophase, which were reported in both premanifest and symptomatic stages of the disease. These alterations correlate with more severe motor and functional impairments [119]. Additionally, sleep inefficiency and increased nighttime awakenings are commonly observed, further exacerbating cognitive and behavioral deterioration [120].

Melatonin was found to have reduced concentrations in HD patients. This reduction may contribute to the sleep disturbances commonly observed in HD [121].

#### 4.3.3. Perspectives and Therapeutic Outlook

The observed disruptions in melatonin secretion patterns in HD patients suggested a potential role for melatonin as both a biomarker and a therapeutic agent. Further clinical research is needed to evaluate whether supplementing with melatonin can effectively enhance restfulness and restore circadian synchronization in individuals diagnosed with HD [122].

In summary, circadian dysregulation and melatonin alterations are prominent features of HD, contributing to the disease’s complex symptomatology. Addressing these disruptions through chronotherapeutic strategies holds promise for improving patient outcomes and quality of life.

### 4.4. Circadian Dysregulation and Melatonin in MS

#### 4.4.1. Preclinical Evidence

Multiple sclerosis (MS) is a long-standing, immune-mediated neurodegenerative condition marked by the loss of myelin sheaths and persistent inflammation within the central nervous system. In preclinical autoimmune demyelination models, melatonin administration was reported to delay disease onset and mitigate demyelination [123].

#### 4.4.2. Clinical Evidence

Circadian misalignment emerged as a significant factor influencing MS onset and exacerbations [124,125]. Additionally, chrononutrition, particularly time-restricted eating (TRE), has the potential to stabilize circadian rhythms and modulate immune responses. TRE aligns food intake with endogenous rhythms, reducing inflammatory cytokine levels and enhancing metabolic regulation [126]. Therefore, integrating melatonin therapy with dietary interventions may provide a comprehensive strategy for managing MS-related circadian dysfunction.

### 4.5. Circadian Dysregulation and Melatonin in ALS

#### 4.5.1. Preclinical Evidence

Amyotrophic lateral sclerosis (ALS) is a rapidly progressive neurodegenerative disease marked by motor neuron degeneration. Although primarily affecting motor pathways, recent evidence revealed substantial circadian involvement in ALS pathology [127].

Disruptions in core clock genes such as *BMAL1* and REV-ERBα were detected in ALS patients and cell-based models. Reduced REV-ERBα expression was associated with increased neuroinflammation, mitochondrial dysfunction, and motor neuron loss in ALS [128]. Melatonin, on the other hand, could offer a promising intervention for ALS. Its administration restores redox balance, enhances mitochondrial biogenesis, and suppresses glial activation in experimental ALS models [129].

#### 4.5.2. Clinical Perspective and Future Outlook

The convergence of circadian gene dysregulation, mitochondrial impairment, and melatonin deficiency underscored the need for integrated chronotherapeutic approaches in ALS [130]. In future studies, personalized regimens combining melatonin supplementation with lifestyle alignment could potentially delay disease progression and support symptomatic management.

### 4.6. Circadian Dysregulation in Psychiatric and Neuropsychiatric Disorders

#### Clinical Evidence

Circadian rhythm disruptions are increasingly recognized as contributing factors to the pathogenesis, onset, and maintenance of psychiatric disorders, particularly mood disorders and schizophrenia. Altered glucocorticoid rhythms related to hypothalamic–pituitary–adrenal (HPA) axis dysfunction, as well as dysregulated melatonin levels, were linked to these conditions. Genetic studies also highlighted abnormalities in core circadian clock genes in psychiatric populations [131,132].

Neuropsychiatric symptoms are now integral to the diagnostic criteria of several neurodegenerative diseases. Syndromes such as apathy, disinhibition, and REM sleep behavior disorders are often present and reflect disruptions in circadian and neuroendocrine regulation [133].

Table 3 summarizes key findings on circadian dysregulation and melatonin disturbances, along with emerging therapeutic strategies in various neurodegenerative and psychiatric conditions.

## 5. Chrononutrition and the Gut–Brain–Clock Axis

### 5.1. Circadian Regulation of the Gut Microbiota

Emerging evidence highlights the bidirectional relationship between circadian rhythms and the gut microbiota, emphasizing their joint role in neurodegenerative disease pathogenesis. The gut microbiome exhibits diurnal oscillations that are regulated by the host’s internal clock, and in turn, short-chain fatty acids (SCFAs) such as butyrate feed back to modulate host circadian gene expression through histone deacetylase inhibition and epigenetic remodeling [134].

Disruptions in circadian rhythms due to aging, sleep deprivation, or high-fat diets lead to gut dysbiosis, characterized by reduced microbial diversity, loss of rhythmic SCFA production, and impaired intestinal barrier function. These changes have been linked to increased neuroinflammatory responses and cognitive impairment in both preclinical and clinical studies [135,136].

In murine models, chronic jet lag alters the composition and diurnal cycling of key microbial taxa, which in turn impairs glucose homeostasis and inflammatory tone [137]. Furthermore, tryptophan-derived microbial metabolites such as indole derivatives regulate melatonin biosynthesis and gut–brain signaling, forming a key node in the circadian–microbiota axis [138]. Thus, restoring microbial oscillations via chrononutrition, timed feeding, or dietary polyphenols not only improves microbiota rhythmicity but also enhances cognitive resilience in aging and neurodegenerative conditions [139].

### 5.2. Melatonin and Intestinal Crosstalk

Melatonin is abundantly produced in the gastrointestinal system and is essential for maintaining the structural integrity of the intestinal barrier while also modulating host–microbiome communication. The gut microbiota contributes to melatonin biosynthesis via tryptophan metabolism, while melatonin reciprocally influences microbial diversity and intestinal permeability [140].

Specifically, indole derivatives such as indole-3-acetaldehyde and indole-3-propionic acid, produced by *Lactobacillus* and *Clostridium* species, serve as key intermediates in melatonin synthesis and have been shown to regulate mucosal immunity and epithelial tight junction integrity [141].

Disruption of this bidirectional interaction, such as in gut dysbiosis, was associated with a decline in melatonin production and the onset of neuroinflammatory states, highlighting a potential link between gut health and neurological function. Moreover, melatonin was reported to exert modulatory effects on circadian gene expression in the gut, which, in turn, can influence microbial rhythmicity and metabolic output [134]. Notably, in aged mice exposed to light-at-night conditions, melatonin supplementation not only restored gut microbial rhythm but also reduced intestinal inflammation and blood–brain barrier permeability [142].

This dynamic interplay forms a crucial component of the microbiota–gut–brain axis and may underlie the pathophysiology of several neurodegenerative conditions. These findings offer translational relevance for therapeutic approaches that target both the circadian and microbial components of neurodegeneration.

### 5.3. Nutrition, Obesity, and Circadian Rhythms

Nutrition acts as a potent zeitgeber, modulating the rhythmic activity of peripheral clocks through the timing, composition, and frequency of food intake. Unlike light, which primarily entrains the central SCN, feeding behaviors directly influence peripheral oscillators, especially in metabolic organs such as the liver, intestine, pancreas, and adipose tissue. Irregular eating patterns, late-night feeding, and high-fat diets were shown to disrupt circadian synchrony by desynchronizing these peripheral clocks from the central pacemaker. This misalignment leads to impaired metabolic regulation, elevated oxidative stress, and systemic inflammation, all of which are critical contributors to neurodegenerative disease progression [143,144].

In both animal models and clinical settings, chrononutritional interventions such as time-restricted eating, early time-restricted feeding, and intermittent fasting were shown to restore rhythmic gene expression, improve metabolic flexibility, and reduce neuroinflammation. These dietary strategies help reestablish temporal alignment between nutrient intake and the endogenous circadian system, enhancing resilience to cognitive decline and neurodegenerative processes [145,146].

Obesity further amplifies circadian disruption through complex bidirectional mechanisms. As an endocrine organ, adipose tissue exhibits its circadian rhythmicity, secreting hormones such as leptin and adiponectin in a time-dependent manner. In obesity, these rhythms are blunted, contributing to disrupted appetite regulation, increased insulin resistance, and chronic low-grade inflammation [147,148]. Moreover, obesity-associated elevations in pro-inflammatory cytokines can affect hypothalamic function and alter the expression of core clock genes in both central and peripheral tissues, thereby exacerbating circadian misalignment [149,150].

Experimental studies investigated that diet-induced obesity impairs hippocampal synaptic plasticity and reduces the amplitude of circadian oscillations in brain regions responsible for learning and memory [151,152,153]. Additionally, clock gene mutant mice spontaneously developed obesity and metabolic syndrome, even under standard diet conditions, due to the loss of diurnal feeding rhythms and decreased expression of circadian and metabolic regulators such as Per2, orexin, and ghrelin [154]. These findings supported the notion that obesity and circadian disruption interact in a feed-forward loop, promoting neuroinflammatory states, energy imbalance, and increased neurodegenerative vulnerability.

## 6. Chronopharmacology

### 6.1. Melatonin Analogs

Synthetic melatonin analogs, including ramelteon, agomelatine, and tasimelteon, were developed to overcome the pharmacokinetic limitations of melatonin, such as low bioavailability and short half-life. These agents demonstrated improved receptor selectivity, longer-lasting effects, and greater therapeutic precision in circadian-related disorders.

Agomelatine acts as a high-affinity agonist at MT1 (Ki ≈ 0.1 nM) and MT2 (Ki ≈ 0.12 nM) receptors and as a selective antagonist at 5-HT_2_C. This dual mechanism enables both circadian resynchronization and antidepressant activity. The 5-HT_2_C antagonism increases dopamine and norepinephrine release in the frontal cortex, a property absent in melatonin [155,156]. Agomelatine is currently approved for major depressive disorder (MDD) in adults under 75 years of age.

Tasimelteon has a higher affinity for MT2 (Ki = 0.069 nM) than MT1 (Ki = 0.304 nM), supporting its role in circadian phase shifting. It is approved for non-24-hour sleep–wake disorder, especially in blind individuals, and exhibits a longer half-life (~1–2 h) than melatonin, with more predictable receptor engagement [156].

Compared to melatonin, these analogs offer greater receptor subtype selectivity, improved bioavailability, and longer pharmacological half-lives, enhancing their clinical utility in specific circadian and psychiatric conditions. Nonetheless, head-to-head trials are needed to evaluate their long-term cognitive and neuroprotective outcomes in neurodegenerative disease populations [156].

While most studies utilized melatonin doses between 40 and 100 mg/day [157], future research should aim at formulation improvements, chronopharmacological timing, and biomarker-guided stratification to fully leverage the therapeutic potential of melatonergic agents.

### 6.2. Drug Timing

The concept of chronopharmacology examines how the timing of drug administration affects pharmacokinetics, pharmacodynamics, and therapeutic efficacy. Biological processes demonstrate circadian rhythms, pharmacokinetic phases, target receptor sensitivity, and downstream signaling pathways [158]. Therefore, aligning medication timing with endogenous circadian rhythms can optimize efficacy and minimize adverse effects.

Several classes of neuroactive drugs demonstrated time-dependent efficacy. For instance, the administration of acetylcholinesterase inhibitors such as donepezil in the morning was associated with improved behavioral outcomes and reduced sleep disturbances in AD patients [159]. Similarly, the timing of monoamine oxidase-B inhibitors like selegiline may influence dopaminergic tone and motor symptom control in PD [160]. Corticosteroids, often used in MS, showed enhanced anti-inflammatory activity and reduced toxicity when dosed in alignment with circadian cortisol rhythms [161].

Chronopharmacology also plays a role in melatoninergic treatments. Melatonin’s efficacy is highly timing-dependent, with administration in the evening or at dim-light melatonin onset (DLMO) producing the greatest phase-shifting effects. Mistimed administration may lead to phase delays or reduced therapeutic impact [162]. Moreover, circadian timing of drug administration influences gene expression profiles; for instance, clock-controlled transcription factors modulate the expression of metabolic enzymes and membrane transporters involved in drug metabolism [163].

In addition to time-of-day-dependent efficacy, potential pharmacokinetic and pharmacodynamic interactions between melatonin and neuroactive agents must be considered. For example, in PD, co-administration of melatonin with L-dopa enhanced dopaminergic response and motor coordination while mitigating oxidative stress, as demonstrated in MPTP-induced parkinsonian mice. This effect was attributed to melatonin’s antioxidant activity and its capacity to preserve striatal dopamine levels by reducing lipid peroxidation and enhancing glutathione peroxidase activity [164]. In AD and mild cognitive impairment, melatonin given alongside cholinesterase inhibitors such as donepezil improved cognitive performance, sleep quality, and reduced depressive symptoms, supporting its use as an adjunctive chronotherapeutic agent [165].

Furthermore, melatonin hybrids were explored in preclinical models as multitarget agents combining antioxidant, cholinesterase-inhibitory, and monoamine oxidase-inhibitory effects, demonstrating promising interactions with donepezil and other agents for synergistic modulation of AD pathophysiology [166].

### 6.3. Emerging Therapeutic Targets

Recent advances in chronobiology and neurodegenerative research identified nuclear receptors and epigenetic regulators as promising targets for therapeutic modulation. Among these, RORα, REV-ERBα, and SIRT1 garnered attention for their pivotal roles in linking circadian timing to cellular metabolism, neuroinflammation, and mitochondrial homeostasis [167].

RORα functions as a transcriptional activator within the auxiliary loop of the circadian clock, positively regulating *BMAL1* expression and enhancing circadian amplitude. In neurodegenerative disease models, RORα activation was investigated to suppress microglial activation, promote anti-inflammatory phenotypes, and protect synaptic integrity. Pharmacological agonists of RORα, such as SR1078, demonstrated efficacy in reducing neuroinflammation and improving behavioral performance in preclinical models of PD and AD [168].

Conversely, REV-ERBα serves as a transcriptional repressor of *BMAL1*, balancing circadian oscillations and contributing to metabolic regulation. Its dysfunction was associated with mitochondrial impairment, elevated oxidative stress, and glial overactivation—key pathomechanisms in AD [169]. Pharmacological activation of REV-ERBα using synthetic ligands such as SR9009 yielded promising results in attenuating neuroinflammatory cascades and restoring circadian rhythmicity [170].

Furthermore, SIRT1 represents a key node between metabolic sensing and epigenetic regulation of circadian genes. SIRT1 deacetylates *BMAL1* and *PER2*, influencing circadian period length and amplitude. It also modulates cellular stress responses, autophagy, and mitochondrial biogenesis [171]. In some models of neurodegeneration, SIRT1 activation via resveratrol or SRT1720 was also linked to enhanced neuronal survival, reduced tau pathology, and improved cognitive outcomes [172]. Collectively, the pharmacological modulation of these molecular targets offers a novel chronotherapeutic axis with the potential to recalibrate disrupted molecular clocks and mitigate disease progression.

### 6.4. Non-Pharmacological Interventions: Music Therapy

Music therapy (MT) had considerable efficacy in improving the quality of life in individuals affected by neurodegenerative conditions, including AD and PD. A study by Sharma et al. proposed that rhythm-based interventions can exert neuroprotective effects by stabilizing circadian rhythms, potentially mitigating disease progression. MT was investigated to influence not only cognitive and emotional domains but also neural circuitry involved in circadian regulation, thus representing a multifaceted non-pharmacological intervention [173]. Nonetheless, there is a need for longitudinal studies that examine the direct effects of MT on sleep architecture and circadian entrainment.

## 7. Conclusions and Future Directions

This review summarizes current findings elucidating the molecular mechanisms underlying the intricate interplay between circadian rhythms, melatonin signaling, and neurodegenerative diseases. Despite robust preclinical evidence and emerging clinical data, the translation of circadian-based strategies into routine clinical practice faces several challenges. These include the variability in individual circadian profiles, lack of standardized biomarkers, and limited large-scale randomized trials evaluating chronotherapy and melatonin analogs in neurodegenerative populations. Moreover, the heterogeneity of disease phenotypes necessitates a personalized medicine approach that integrates circadian assessment tools, biomarker-based stratification, and individualized treatment timing.

Future research should first prioritize the development of robust and clinically applicable circadian biomarkers. Peripheral clock gene expression, salivary melatonin profiles, and actigraphy-derived rest–activity patterns hold potential for early diagnosis, disease staging, and response monitoring. The validation of these markers across diverse populations will enable biomarker-driven stratification in both observational and interventional studies.

The integration of wearable technologies and machine learning tools will play a crucial role in enabling real-time circadian profiling. These digital platforms can track individual rhythmicity and generate predictive models for symptom progression and optimal treatment timing. The fusion of bioinformatics and chronobiology is expected to yield precision-based therapeutic frameworks in the future.

The pharmacological modulation of circadian regulators such as RORα, REV-ERBα, and SIRT1 represents a promising therapeutic axis. These targets are tightly linked to key disease mechanisms, including neuroinflammation, mitochondrial dysfunction, and epigenetic dysregulation. Future studies should investigate the efficacy and safety of small-molecule agonists or antagonists of these regulators in neurodegenerative disease models.

Finally, there is a strong rationale for exploring multimodal interventions that combine pharmacological agents (e.g., melatonin or its analogs) with non-pharmacological strategies, including light therapy, time-restricted feeding, and music-based entrainment. These approaches may enhance circadian coherence and offer synergistic benefits for both motor and cognitive symptoms.

Alongside therapeutic innovations, future research should consider the sex-specific differences in circadian biology and treatment response warrant greater attention to ensure more personalized and effective chronotherapeutic strategies.

## Figures and Tables

**Table 1:** Central and peripheral circadian regulators and core clock gene functions in TTFLs.

Component	Location	Function	Mechanism/Pathway	Notes/Role in Rhythm Maintenance	Ref(s)
SCN	Anterior hypothalamus	Master circadian pacemaker	Receives light via retinohypothalamic tract → CREB activation → clock gene induction	Synchronizes peripheral clocks, modulates neuroendocrine and behavioral rhythms	[20,21,22]
Peripheral Clocks	All tissues	Local timekeeping	Follows TTFL but influenced by feeding, temperature, and glucocorticoids	Depends on SCN for phase alignment	[23]
CLOCK	Nucleus	Transcriptional activator	Dimerizes with BMAL1 → binds E-box → activates Per/Cry	Positive arm of TTFL	[24]
BMAL1	Nucleus	Core transcription factor	Forms CLOCK–BMAL1 complex → initiates circadian transcription	Essential for rhythm generation	[24]
PER (PER1–3)	Cytoplasm Nucleus	Transcriptional repressors	Phosphorylated by CK1δ/ε → translocates to nucleus → inhibits CLOCK–BMAL1	Forms negative arm of feedback loop	[25]
CRY (CRY1/2)	Cytoplasm Nucleus	Transcriptional repressors	Stabilizes PER, part of the inhibitory complex	Completes core loop with PER	[25]
CK1δ/ε	Cytoplasm	Kinase	Phosphorylates PER/CRY → regulates degradation and nuclear entry	Controls circadian period length	[25]
REV-ERBα	Nucleus	Transcriptional repressor	Binds RRE in BMAL1 promoter → represses BMAL1 transcription	Stabilizes loop amplitude	[24,25]
RORα	Nucleus	Transcriptional activator	Binds the same RRE as REV-ERBα → promotes BMAL1 transcription	Antagonistic to REV-ERBα, maintains phase precision	[24,25]

Notes: TTFL: transcriptional–translational feedback loop; SCN: suprachiasmatic nucleus; CLOCK: circadian locomotor output cycles kaput; BMAL1: brain and muscle ARNT-like protein-1; PER: period; CRY: cryptochrome; CK1δ/ε: casein kinase 1 delta/epsilon; REV-ERBα: nuclear receptor subfamily 1 group D member 1; RORα: retinoic acid-related orphan receptor alpha; RRE: ROR response element; E-box: enhancer box (a DNA promoter element); CREB: cAMP response element-binding protein.

**Table 2:** Preclinical models and molecular mechanisms underlying the protective effects of melatonin on the BBB.

Model/System	Model Type/Species	Target/Pathway	Molecular Mechanism	Observed Effect on BBB	Reference(s)
Methamphetamine-induced toxicity	In vivo, Rat	NADPH oxidase 2	Inhibition via melatonin receptors	Prevention of endothelial damage	[45,46,47,48]
Excitotoxic insult via ibotenate	In vivo, Neonatal Rat	Occludin, Claudin-5, JAM1/2, ZO-1, Cdh5, ABCG2	Normalization of TJ and transporter expression; prevention of tight junction disruption	Reduced dextran leakage, preserved BBB integrity, smaller lesion size	[49]
Transient focal ischemia	In vivo, Young Mice	Claudin-5, ZO-1	Preservation of tight junction integrity under ischemic stress	Attenuated vascular leakage, reduced infarct area	[50]
BBB tight junction culture model	In vitro, Human endothelial cells	Claudin-5, ZO-1, Occludin	Upregulation of tight junction proteins	Enhanced structural integrity of BBB	[51]
SARS-CoV-2 neuroinvasion model	In vivo and in vitro, Mice and Human Cells	ACE2	Inhibition of ACE2-mediated viral entry	Decreased neuroinvasion, reduced Aβ-related burden	[52]
Gastric cancer cell line (MMP activity)	In vitro, Human cells	MMPs (e.g., MMP-9)	Inhibition of extracellular matrix degradation	Improved BBB stability	[53,54]
LPS-induced neuroinflammation	In vivo, Aged Mice	AMPK pathway, endothelial TJ proteins	AMPK activation reduces inflammation and stabilizes tight junctions	Restored BBB integrity under inflammatory conditions	[55,56,57,58]

Notes: BBB: blood–brain barrier; TJ: tight junction; ZO-1: zonula occludens-1; JAM1/2: junctional adhesion molecule 1/2; Cdh5: cadherin-5; ABCG2: ATP-binding cassette sub-family G member 2; AMPK: AMP-activated protein kinase; MMP: matrix metalloproteinase; ACE2: angiotensin-converting enzyme 2; Aβ: amyloid-beta; LPS: lipopolysaccharide.

**Table 3:** Circadian dysregulation, melatonin alterations, and therapeutic insights across neurodegenerative and psychiatric disorders.

Disease	Circadian Disturbance	Melatonin Alteration	MT1/MT2-Linked Pathways	Molecular/Pathway Involvement	Preclinical/Clinical Evidence	Therapeutic Implications	Reference(s)
AD	Fragmented sleep, blunted circadian markers	↓ CSF/plasma melatonin, especially at night	MT1 → PI3K/AKT → GSK3β ↓ MT2 → SIRT1 ↑, AQP4 ↑	BDNF ↓, SIRT1, PERK, AQP4, MTNR1A/B polymorphisms	294 rodent studies: ↑ CREB/BDNF, cognitive improvement; RCTs: improved MMSE, sleep	Melatonin + light therapy may improve sleep and agitation	[78,79,80,81,82,83,84,85,86,87,88,89,90,91,92,93,94,95,96,97,98,99,100,101,102,103,104]
PD	Reduced amplitude, disrupted SCN, iRBD	↓ Nocturnal melatonin, REM loss	MT2 → Nrf2 ↑, REV-ERBα ↑ MT1/MT2 → cytokine suppression	Nrf2, REV-ERBα, pro-inflammatory cytokines	Melatonin improves RBD and motor/REM sleep in trials	Potential adjunctive chronotherapy needs dosage standardization	[108,109,110,111,112,113,114,115,116]
HD	Delayed melatonin onset, sleep fragmentation	↓ Melatonin secretion in early and late stages	MT1 → PER/CRY regulation, circadian resynchronization	Circadian gene dysregulation, SCN damage	Drosophila models: circadian rescue, behavioral benefit	Possible biomarker and therapeutic for rhythm restoration	[117,118,119,120,121,122]
MS	Disrupted rhythms, seasonal onset patterns	Altered melatonin; ↓ linked to inflammation	MT1/MT2 → SIRT1 ↑, AMPK ↑, cytokine suppression	Melatonin modulates immune pathways in EAE	Delayed onset, ↓ demyelination with melatonin	Combined with time-restricted feeding (TRE), it may enhance the effect	[123,124,125,126]
ALS	BMAL1, REV-ERBα dysregulation	Melatonin deficiency contributes to redox imbalance	MT1/MT2 → REV-ERBα ↑, SIRT1 ↑, mitochondrial biogenesis	↑ Neuroinflammation, mitochondrial dysfunction	Melatonin: ↑ mitochondrial biogenesis, ↓ glial activation	Chronotherapy may aid disease management	[127,128,129,130]
Psychiatric/Neuropsychiatric Disorders	Abnormal sleep–wake patterns, REM behavior disorder	Dysregulated melatonin rhythms	MT1/MT2 → HPA axis modulation, circadian gene regulation	HPA axis, glucocorticoids, circadian gene variants	Linked to mood disorders, schizophrenia; RBD in neurodegeneration	Potential for rhythm-based interventions	[131,132,133]

Notes: ↓ = decreased; ↑ = increased; SCN: suprachiasmatic nucleus; iRBD: isolated REM sleep behavior disorder; CSF: cerebrospinal fluid; MMSE: Mini-Mental State Examination; BDNF: brain-derived neurotrophic factor; SIRT1: sirtuin 1; PERK: protein kinase RNA-like ER kinase; AQP4: aquaporin-4; MTNR1A/B: melatonin receptor 1A/1B (also known as MT1/MT2); PI3K/AKT: phosphatidylinositol 3-kinase/protein kinase B pathway; GSK3β: glycogen synthase kinase 3 beta; Nrf2: nuclear factor erythroid 2–related factor 2; REV-ERBα: nuclear receptor subfamily 1, group D, member 1; AMPK: AMP-activated protein kinase; HPA axis: hypothalamic–pituitary–adrenal axis; TRE: time-restricted eating; EAE: experimental autoimmune encephalomyelitis; RBD: REM sleep behavior disorder.

## Abbreviations

The following abbreviations are used in this manuscript:

AANAT	Arylalkylamine N-Acetyltransferase
AD	Alzheimer’s Disease
AKT	Protein Kinase B
ALAN	Artificial Light at Night
ALS	Amyotrophic Lateral Sclerosis
AMPK	AMP-Activated Protein Kinase
AQP-4	Aquaporin-4
ASMT	Acetylserotonin O-Methyltransferase
AVP	Arginine Vasopressin
Aβ	Amyloid-Beta
BBB	Blood–Brain Barrier
BDNF	Brain-Derived Neurotrophic Factor
BMAL1	Brain and Muscle ARNT-Like 1
CCG	Clock-Controlled Genes
CK1δ/ε	Casein Kinase 1 Delta/Epsilon
CLOCK	Circadian Locomotor Output Cycles Kaput
CREB	cAMP Response Element-Binding Protein
CRY	Cryptochrome (gene family)
CSF	Cerebrospinal Fluid
DLB	Dementia with Lewy Bodies
DLMO	Dim-Light Melatonin Onset
GSK3β	Glycogen Synthase Kinase-3 Beta
HD	Huntington’s Disease
HIOMT	Hydroxyindole-O-Methyltransferase
HPA	Hypothalamic–Pituitary–Adrenal (axis)
ipRGC	Intrinsically Photosensitive Retinal Ganglion Cells
MDD	Major Depressive Disorder
MMP	Matrix Metalloproteinase
MMSE	Mini-Mental State Examination
MoCA	Montreal Cognitive Assessment
MPTP	1-Methyl-4-phenyl-1,2,3,6-tetrahydropyridine
MS	Multiple Sclerosis
MT	Music Therapy
MT1	Melatonin Receptor 1A
MT2	Melatonin Receptor 1B
NLRP3	NOD-like Receptor Protein 3
PD	Parkinson’s Disease
PER	Period (gene family)
PGC-1α	Peroxisome Proliferator-Activated Receptor Gamma Coactivator 1-Alpha
PI3K	Phosphatidylinositol 3-Kinase
RBD	REM Sleep Behavior Disorder
REV-ERBα	Reverse-Erb Alpha
RORα	Retinoic Acid Receptor-Related Orphan Receptor Alpha
ROS	Reactive Oxygen Species
RRE	ROR Response Element
SCFA	Short-Chain Fatty Acids
SCN	Suprachiasmatic Nucleus
SIRT1	Sirtuin 1
TRE	Time-Restricted Eating
TTFL	Transcriptional–Translational Feedback Loop
VIP	Vasoactive Intestinal Peptide
